# Functional characterization and differential nutritional regulation of putative Elovl5 and Elovl4 elongases in large yellow croaker (*Larimichthys crocea*)

**DOI:** 10.1038/s41598-017-02646-8

**Published:** 2017-05-23

**Authors:** Songlin Li, Óscar Monroig, Tianjiao Wang, Yuhui Yuan, Juan Carlos Navarro, Francisco Hontoria, Kai Liao, Douglas R. Tocher, Kangsen Mai, Wei Xu, Qinghui Ai

**Affiliations:** 1 Key Laboratory of Aquaculture Nutrition and Feed (Ministry of Agriculture) and Key Laboratory of Mariculture (Ministry of Education), Ocean University of China, Qingdao, 266003 People’s Republic of China; 2 Institute of Aquaculture, School of Natural Sciences, University of Stirling, Stirling, FK9 4LA Scotland UK; 3 Instituto de Acuicultura Torre de la Sal (IATS-CSIC), Ribera de Cabanes, 12595 Castellón Spain; 4 Laboratory for Marine Fisheries and Aquaculture, Qingdao National Laboratory for Marine Science and Technology, Qingdao, 266003 People’s Republic of China

## Abstract

In the present study, two elongases, Elovl4 and Elovl5, were functionally characterized and their transcriptional regulation in response to n-3 LC-PUFA administration were investigated *in vivo* and *in vitro*. We previously described the molecular characterization of croaker *elovl5*. Here, we report the full-length cDNA sequence of croaker *elovl4*, which contained 1794 bp (excluding the polyA tail), including 909 bp of coding region that encoded a polypeptide of 302 amino acids possessing all the characteristic features of Elovl proteins. Functional studies showed that croaker Elovl5, displayed high elongation activity towards C_18_ and C_20_ PUFA, with only low activity towards C_22_ PUFA. In contrast, croaker Elovl4 could effectively convert both C_20_ and C_22_ PUFA to longer polyenoic products up to C_34_. n-3 LC-PUFA suppressed transcription of the two elongase genes, as well as *srebp*-*1* and *lxrα*, major regulators of hepatic lipid metabolism. The results of dual-luciferase reporter assays and *in vitro* studies both indicated that the transcriptions of *elovl5* and *elovl4* elongases could be regulated by Lxrα. Moreover, Lxrα could mediate the transcription of *elovl4* directly or indirectly through regulating the transcription of *srebp*-*1*. The above findings contribute further insight and understanding of the mechanisms regulating LC-PUFA biosynthesis in marine fish species.

## Introduction

Long chain (C_20–24_) polyunsaturated fatty acids (LC-PUFA), especially the omega-3 (n-3) fatty acids eicosapentaenoic acid (20:5n-3, EPA) and docosahexaenoic acid (22:6n-3, DHA), play pivotal roles in promoting cognitive development, enhancing immune function and minimizing the risk of cardiovascular disease^1–3^. The limited biosynthetic capacity for LC-PUFA in humans makes consumption of fish, especially oily fish, a key component of the diet to guarantee adequate supply of these physiologically essential nutrients^4^. Farmed fish that now contribute significantly to seafood intake in the human diet have been traditionally produced with diets containing high levels of marine fishmeal (FM) and fish oil (FO), ingredients that ensured good growth rates and high levels of n-3 LC-PUFA in the flesh. However, the ever-increasing use of vegetable oils (VO) in aquafeeds has led to reduced deposition of n-3 LC-PUFA in farmed fish and, consequently, to a decrease in their nutritional value for human consumers^4–6^. This has prompted interest in elucidating the mechanisms underlying the endogenous LC-PUFA biosynthetic pathways in farmed finfish species^7, 8^.

Elongases of very long-chain fatty acids (Elovl) catalyze the rate-limiting condensation step in the elongation of fatty acids including LC-PUFA biosynthesis^9, 10^. The crucial role of Elovl enzymes has also been confirmed in fish species^7, 11^, and the overexpression of fish elongases elevated the endogenous production of LC-PUFA in transgenic zebrafish and nibe croaker^12, 13^. Three fatty acid elongases, Elovl2, Elovl4 and Elovl5, have been identified and functionally characterized as crucial enzymes involved in the biosynthetic pathway of LC-PUFA^11^. While Elovl2 appears to be lost in Acanthopterygii, a phylogenic group that includes the vast majority of the most important farmed marine fish species, Elovl4 and Elovl5 are present in virtually all fish species^11^. Importantly, functional studies have shown that Elovl5 can effectively elongate both C_18_ and C_20_ PUFA, whereas Elovl4 is mainly involved in the elongation of C_20–22_ LC-PUFA producing polyenes up to 36 carbons^7, 11^.

Dietary fatty acids can regulate the transcription of *elovl5* and *elovl4* in fish and, generally, expression of these elongase genes is suppressed by dietary n-3 LC-PUFA^14–20^. Additionally, PUFA and their metabolites regulate lipid metabolism, including inhibition of lipogenesis and activation of fatty acid oxidation, through several transcription factors such as the nuclear receptors liver X receptor α (Lxrα) and sterol regulatory element-binding proteins (Srebp)^21^. Srebp-1c can stimulate expression of target genes directly through binding to sterol response elements (SREs) in the promoter region of target genes^22^. In addition to the direct regulation mechanism, Lxrα can also indirectly mediate the expression of target genes through regulating the transcription of *srebp*-*1* and certain other transcription factors^23^. However, the role of these transcription mediators on the expression of elongases Elovl4 and Elovl5 remains poorly understood in fish species. It has been demonstrated that grouper *elovl5* promoter activity was elevated by over-expression of *lxrα*, but not when *srebp*-*1* was over-expressed, indicating the direct regulation role of Lxrα on *elovl5* expression^16^. However, mouse *elovl5* was activated by Srebp-1c directly, and also by Lxrα indirectly elevating the transcription of *elovl5* via Srebp-1c^24^. Elovl4 is the member of Elovl family that has been investigated most recently in fish and no information is available on the underlying regulation mechanism of *elovl4* ^25^.

Large yellow croaker (*Larimichthys crocea*) is an important carnivorous marine fish species widely cultured in southeast China. Recently, low retention of n-3 LC-PUFA has been observed after a large proportion of dietary FO was replaced by VO, which seriously affected the nutritional quality of the fillet of farmed croaker^26^. Recently an *elovl5*-like gene (cDNA) was cloned and its mRNA expression was investigated in response to dietary fatty acids, although no functional data was reported^20^. The aims of the present study were to characterize the function of the previously cloned *elovl5*, and the molecular cloning and functional characterization of an *elovl4* cDNA from *L*. *crocea*. In addition, we also investigated the underlying mechanisms regulating the expression of these two elongases. The results may contribute to increased insight into the potential regulatory mechanisms involved, which will be key to understanding and applying strategies for enhancing the biosynthesis of LC-PUFA in large yellow croaker.

## Results

### Molecular cloning and phylogenetics of the *L*. *crocea* Elovl4

The full-length cDNA of croaker *elovl4* encompassed 1794 bp including a 262 bp 5′ untranslated terminal region (UTR), a 909 bp coding region that encoded 302 aa and a 623 bp 3′ UTR (GenBank Accession No. KP681700). BLAST analysis revealed that the croaker *elovl4* shared high sequence identity with *elovl4* sequences from other teleosts including the orange-spotted grouper (*Epinephelus coioides*, 94%), rabbitfish (*Siganus canaliculatus*, 94%), cobia (*Rachycentron canadum*, 92%) and Atlantic salmon (*Salmo salar*, 86%). Additionally, the croaker *elovl4* cDNA was 40% identical to croaker *elovl5*.

**Figure 1:**
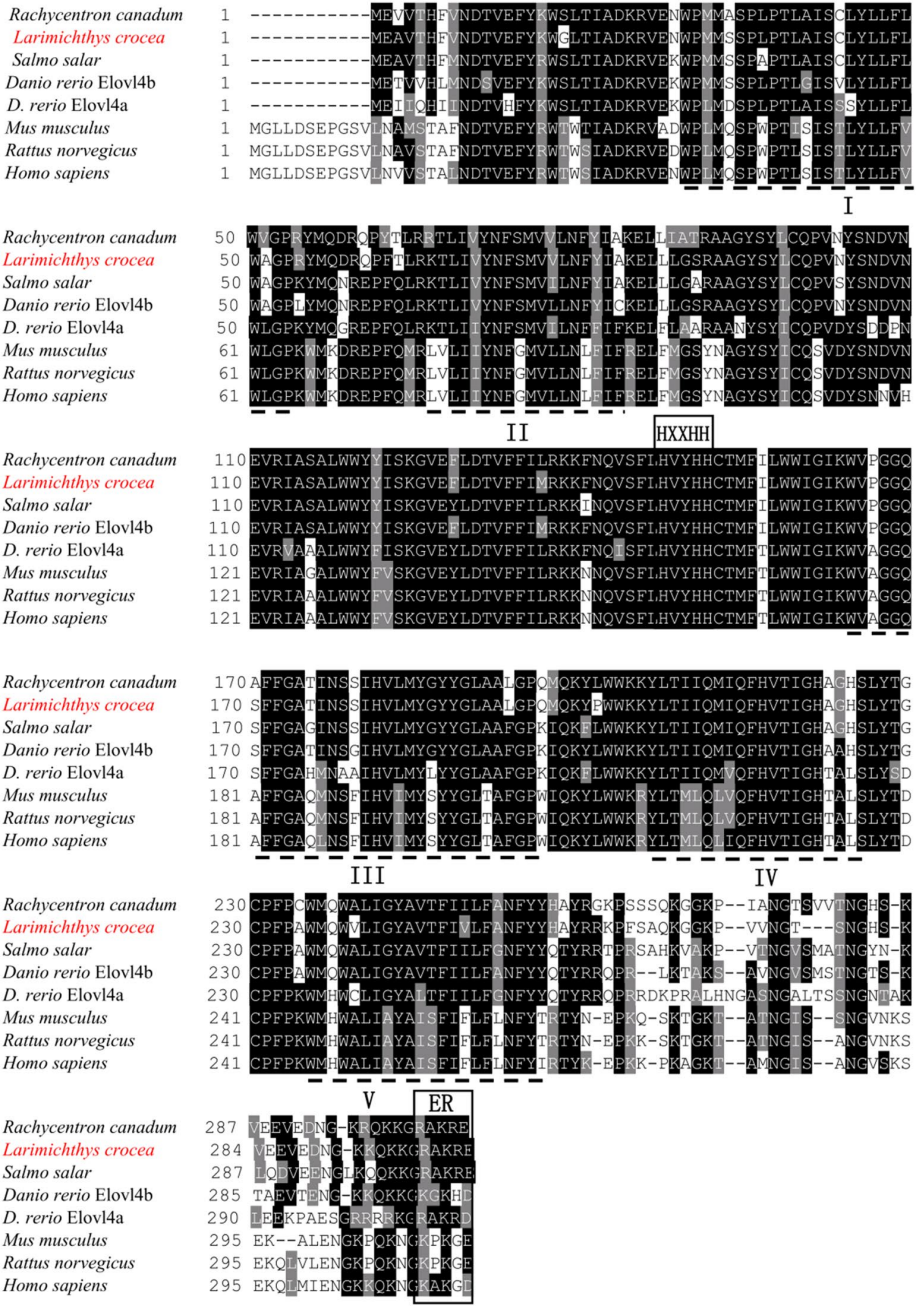
Comparison of the deduced amino acid (AA) sequences of Elovl4 from large yellow croaker, other fish, mouse and human. The AA sequences were aligned using ClustalX, and identity/similarity shading was based on a 75% identity threshold. Identical residues are shaded black and similar residues are shaded grey. Indicated are the conserved HXXHH histidine box motif, five (I–V) putative membrane-spanning domains and the ER retrieval signal.

**Figure 2:**
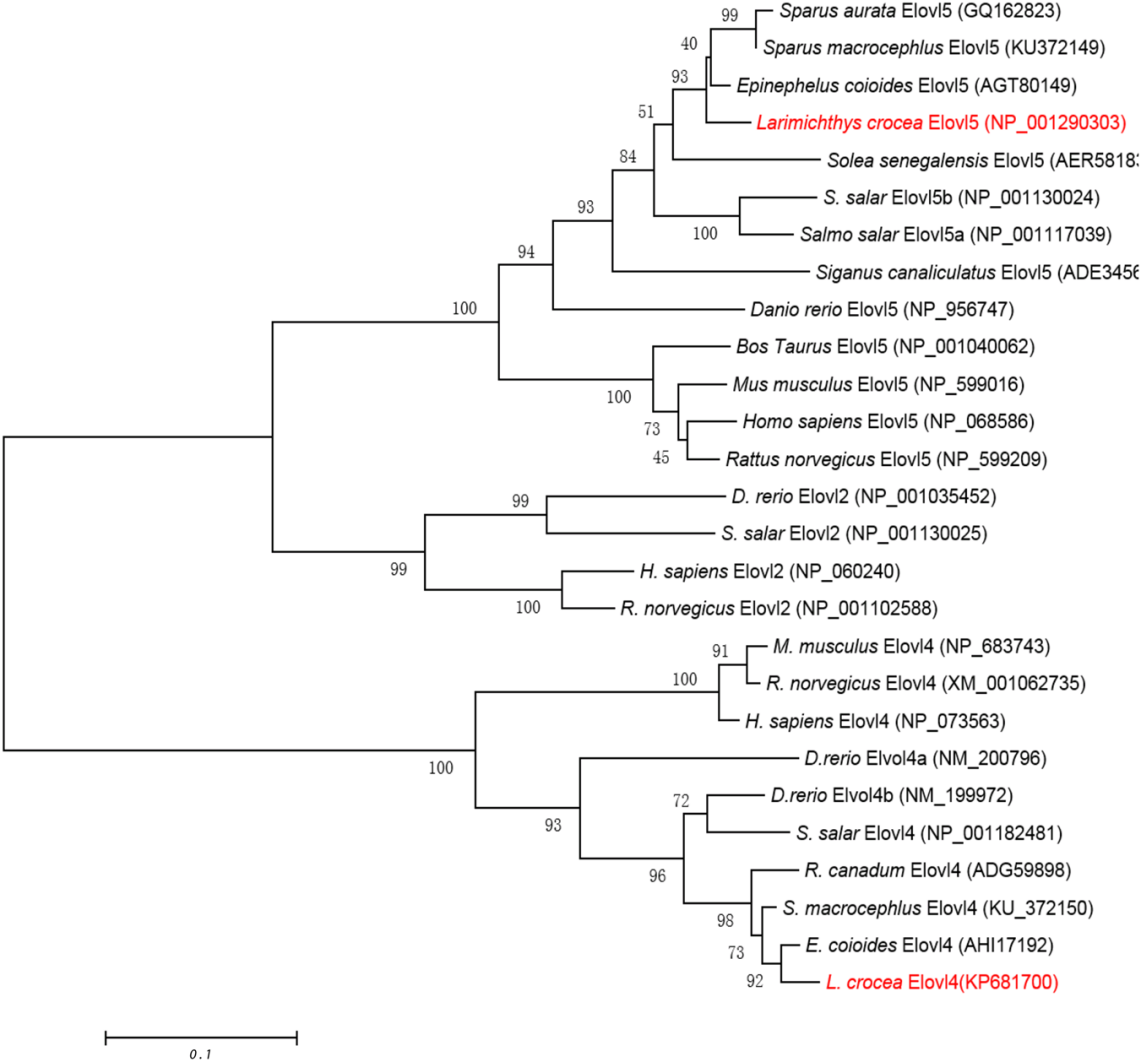
Phylogenetic tree comparing the large yellow croaker Elovl4 with elongase proteins from other organisms. The tree was constructed using the Neighbor Joining method [45] using MEGA4. The numbers represent the frequencies (%) with which the tree topology presented was replicated after 1000 iterations.

Characteristically, the croaker *elovl4* deduced protein contained one histidine box (HXXHH), five putative membrane-spanning domains, and single lysine and arginine residues (RXKXX) at the carboxyl terminus (Fig. 1). Phylogenetic tree analysis showed that the croaker Elovl4 clustered with several other Elovl4-like sequences from fish and mammals, and more distantly with Elovl2 and Elovl5 from these vertebrate lineages (Fig. 2). The results strongly suggested that the newly cloned cDNA encoded an Elovl4 elongase.

### Functional characterization of the Elovl4 and Elovl5 elongases from *L*. *crocea*

The functions of croaker Elovl4 and Elovl5 were characterized by determining the FA profiles of *S*. *cerevisiae* transformed with pYES2-Elo4 or pYES2-Elo5, respectively, and grown in the presence of potential FA substrates. For Elovl4, its role in the biosynthesis of saturated VLC-FA was studied by comparing the saturated FA (>C_24_) profiles of yeast transformed with pYES2-Elo4 and pYES2 (control) (Table 1). No significant differences were found in the content of 24:0, 26:0, 28:0, 30:0 or 32:0, indicating that the croaker Elovl4 does not have a role in the biosynthesis of saturated VLC-FA. In order to investigate the role of the *L*. *crocea* Elovl4 in the biosynthesis of VLC-PUFA, pYES2-Elo4 transformed yeast were grown in the presence of C_18_ (18:4n-3 and 18:3n-6), C_20_ (20:5n-3 and 20:4n-6) and C_22_ (22:5n-3, 22:4n-6 and 22:6n-3) substrates. The results confirmed that croaker Elovl4 was able to elongate all PUFA substrates producing in most cases polyenes of up to 32 or 34 carbons, except for DHA that was only elongated to 24:6n-3 (Table 2). Interestingly, the elongation towards C_28_ and C_30_ substrates showed the highest conversion values (%) regardless of the initial PUFA substrate. However, despite limited activity towards DHA, the croaker Elovl4 could elongate both 20:5n-3 and 22:5n-3 to 24:5n-3, the C_24_ substrate for DHA synthesis.

**Table 1:** Functional characterization of the large yellow croaker Elovl4: Role in biosynthesis of very long-chain saturated fatty acids (FA).

FA	Control	Elovl4
24:0	8.4 ± 2.2	10.8 ± 2.0
26:0	79.4 ± 7.0	78.3 ± 4.9
28:0	7.8 ± 1.7	6.8 ± 3.0
30:0	3.7 ± 2.4	3.4 ± 0.5
32:0	0.6 ± 0.6	0.7 ± 0.7
34:0	nd	nd

Results are expressed as an area percentage of total saturated FA ≥ C_24_ found in yeast transformed with either the empty pYES2 vector (Control) or the large yellow croaker *elovl4* ORF. Results are means ± standard deviations (N = 3). No statistical differences were observed between treatments (Students *t*-test, *P* ≤ 0.05).

nd, not detected.

**Table 2:** Functional characterization of the large yellow croaker Elovl4: conversions of polyunsaturated fatty acid (FA) substrates.

FA substrate	Product	% Conversion
18:4n-3	20:4n-3	8.5
	22:4n-3	32.6
	24:4n-3	71.5
	26:4n-3	100.0
	28:4n-3	100.0
	30:4n-3	94.5
	32:4n-3	72.2
18:3n-6	20:3n-6	6.1
	22:3n-6	36.4
	24:3n-6	67.1
	26:3n-6	90.7
	28:3n-6	93.2
	30:3n-6	89.7
	32:3n-6	21.9
20:5n-3	22:5n-3	14.7
	24:5n-3	47.0
	26:5n-3	69.2
	28:5n-3	92.7
	30:5n-3	98.2
	32:5n-3	81.9
	34:5n-3	11.1
20:4n-6	22:4n-6	21.1
	24:4n-6	50.7
	26:4n-6	65.7
	28:4n-6	85.0
	30:4n-6	91.4
	32:4n-6	41.1
22:5n-3	24:5n-3	23.5
	26:5n-3	59.8
	28:5n-3	90.4
	30:5n-3	97.6
	32:5n-3	75.5
22:4n-6	24:4n-6	35.8
	26:4n-6	71.0
	28:4n-6	89.7
	30:4n-6	94.2
	32:4n-6	49.4
22:6n-3	24:6n-3	1.2

Conversions were calculated for each stepwise elongation according to the formula [areas of first product and longer chain products/(areas of all products with longer chain than substrate + substrate area)] × 100. The substrate FA varies as indicated in each step-wise elongation.

The function of *L*. *crocea* Elovl5 was characterized by growing the transgenic yeast expressing its coding region in the presence of C_18_ (18:3n-3, 18:2n-6, 18:4n-3 and 18:3n-6), C_20_ (20:5n-3 and 20:4n-6) and C_22_ (22:5n-3 and 22:4n-6) PUFA substrates. The results from the yeast assays revealed that the *L*. *crocea* Elovl5 exhibited high conversion towards C_18_ PUFA, particularly 18:4n-3 (86.7%) and 18:3n-6 (77.9%), followed by C_20_ substrates including 20:5n-3 (74.6%) and 20:4n-6 (70.0%). The *L*. *crocea* Elovl5 showed relatively low activity towards C_22_ substrates such as 22:5n-3 (7.6%) and 22:4n-6 (2.3%) (Table 3). Fatty acid composition of yeast transformed with the empty pYES2 vector and grown in the presence of PUFA substrates used in both Elovl4 and Elovl5 functional assays primarily consisted of the four major endogenous FA, namely 16:0, 16:1n-7, 18:0 and 18:1 (including 18:1n-9 and 18:1n-7 isomers), together with any exogenously added FA (data not shown).

**Table 3:** Functional characterization of the large yellow croaker Elovl5: conversions of polyunsaturated fatty acid (FA) substrates.

FA substrate	FA product	% Conversion
18:3n-3	20:3n-3	48.3
	22:3n-3	6.3
	24:3n-3	7.4
18:2n-6	20:2n-6	24.1
	22:2n-6	6.9
18:4n-3	20:4n-3	86.7
	22:4n-3	28.0
	24:4n-3	3.1
18:3n-6	20:3n-6	77.9
	22:3n-6	21.8
	24:3n-6	4.8
20:5n-3	22:5n-3	74.6
	24:5n-3	11.1
	26:5n-3	6.2
20:4n-6	22:4n-6	70.0
	24:4n-6	4.6
	26:4n-6	4.8
22:5n-3	24:5n-3	7.6
	26:5n-3	6.3
22:4n-6	24:4n-6	2.3

Conversions were calculated for each stepwise elongation according to the formula [areas of first product and longer chain products/(areas of all products with longer chain than substrate + substrate area)] × 100.

### Tissue distribution of *elovl4*

**Figure 3:**
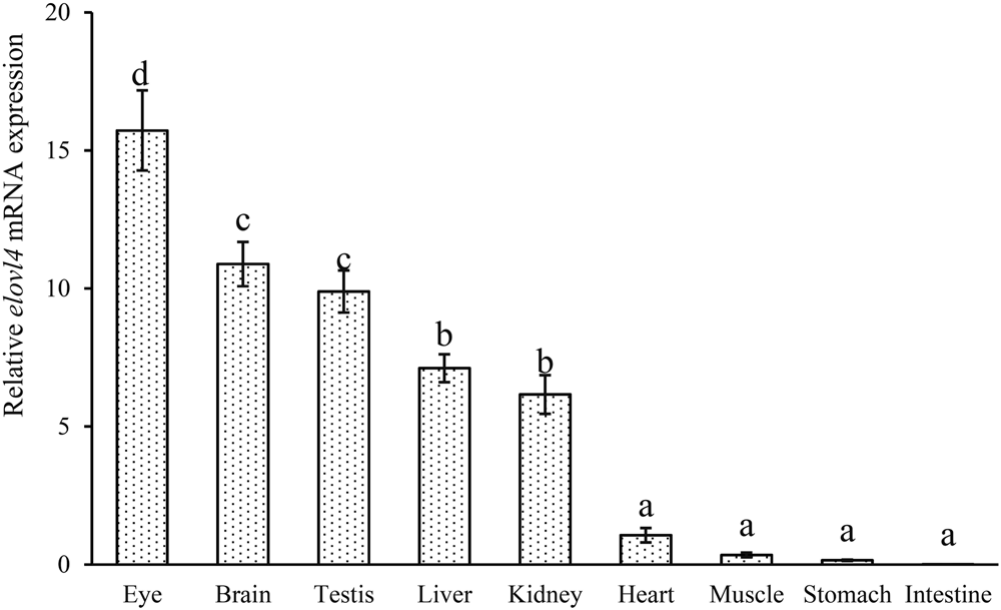
Tissue expression of elovl4 in large yellow croaker. Values (means ± standard error of the mean, SEM) in bars that have the same letter are not significantly different (*P* > 0.05; Tukey’s test) among treatments (n = 3).

The distribution of *elovl4* mRNA in *L*. *crocea* was detected in several tissues, including eye, brain, testis, heart, liver, kidney, spleen, stomach, intestine and muscle. Gene expression analysis revealed that the highest levels of *elovl4* transcript were found in eye, follow by brain and testis (Fig. 3).

### Nutritional regulation of the *L*. *crocea elovl4* and *elovl5* and transcription factors: *in vivo* and *in vitro* trials

**Figure 4:**
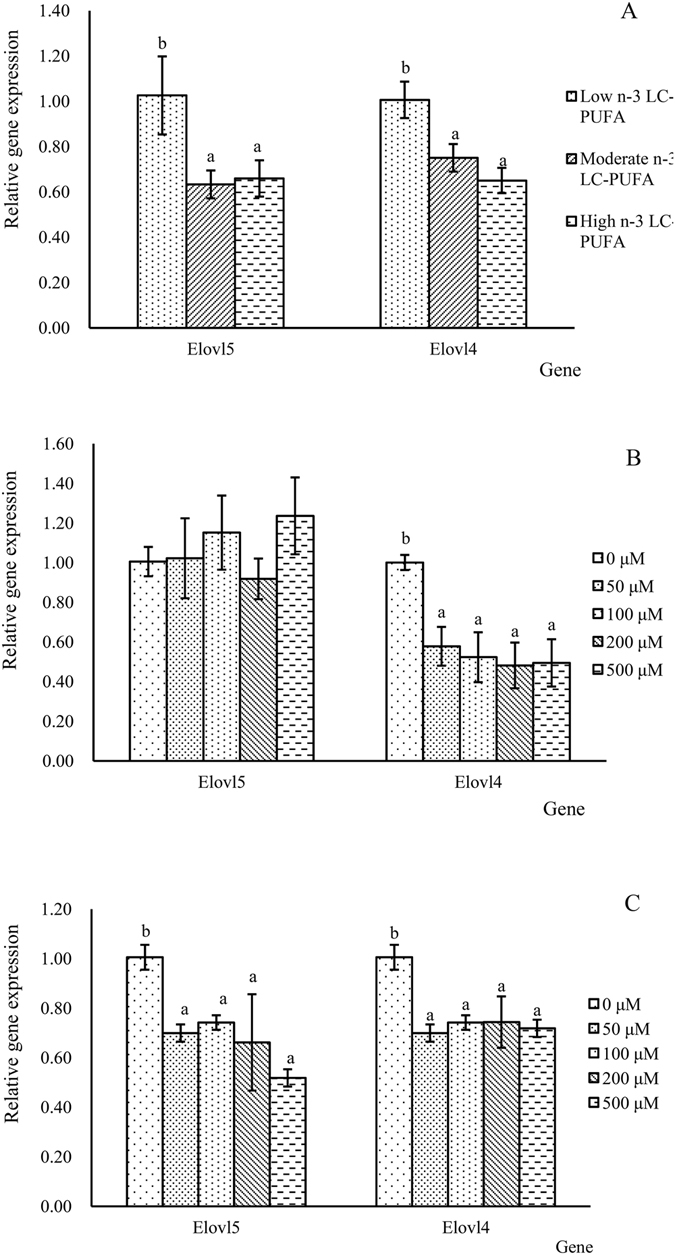
Effects of dietary n-3 LC-PUFA (**A**), DHA (**B**) and EPA (**C**) contents on the expression of *elovl4* and *elovl5*. Values (means ± SEM) in bars that have the same letter are not significantly different (*P* > 0.05; Tukey’s test) among treatments (n = 3).

The expression of *elovl4* and *elovl5* from fish fed the experimental diets containing varying levels of LC-PUFA is shown in Fig. 4A. The results showed that the expression of both genes was down-regulated in fish fed diets containing both high and moderate n-3 LC-PUFA levels in comparison to diets with low n-3 LC-PUFA (*P* < 0.05). *In vitro* assays performed with hepatocyte primary cultures prepared from fish fed a standard diet indicated that the expression of *elovl4*, but not *elovl5*, was down-regulated by DHA supplementation (*P* < 0.05) (Fig. 4B). Additionally, supplementation of EPA to hepatocytes suppressed the expression of both elongases (*P* < 0.05) (Fig. 4C).

**Figure 5:**
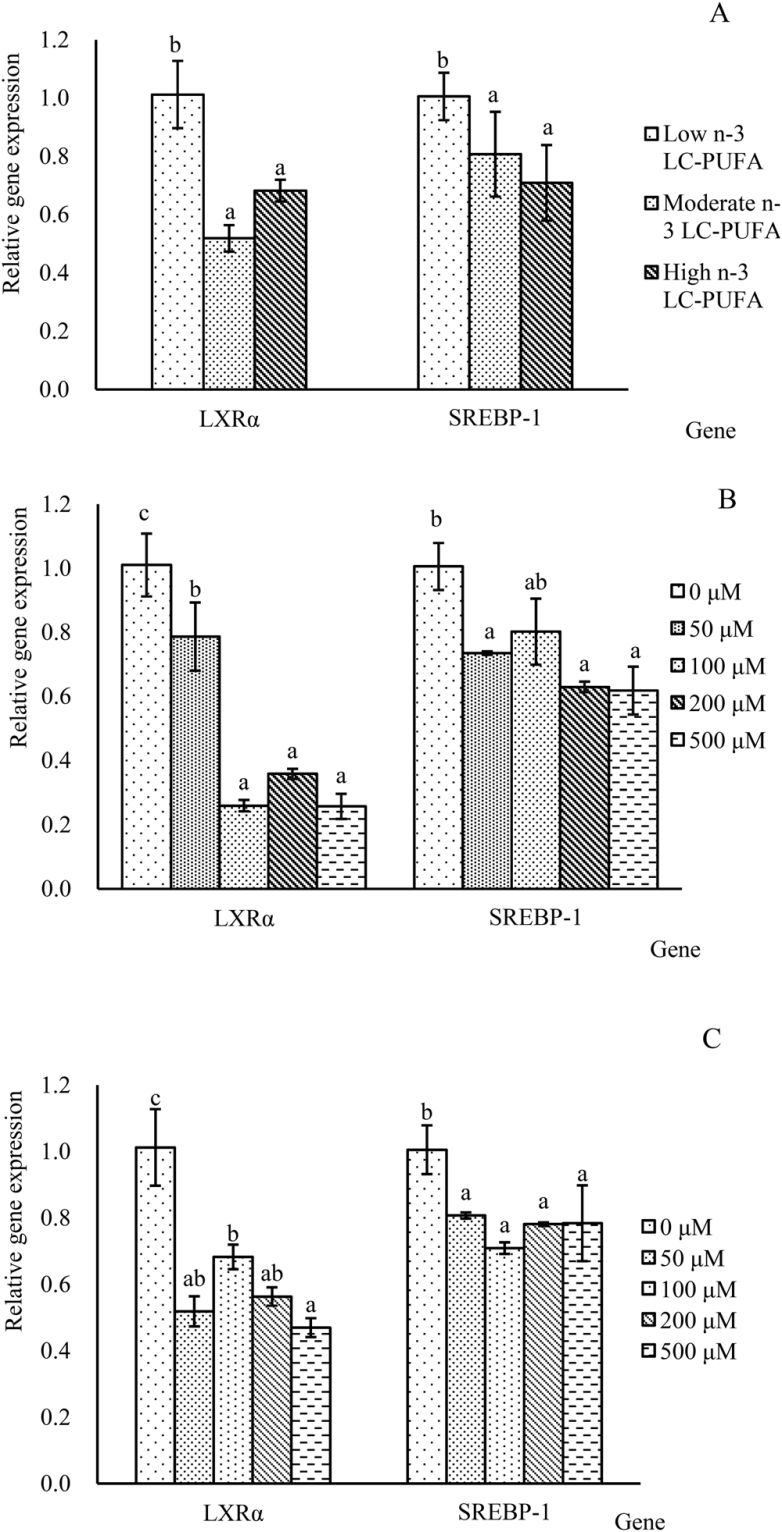
Effects of dietary n-3 LC-PUFA (**A**), DHA (**B**) and EPA (**C**) contents on the expression of the transcription factors *lxrα* and *srebp*-*1*. Values (means ± standard error of the mean, SEM) in bars that have the same letter are not significantly different (*P* > 0.05; Tukey’s test) among treatments (n = 3).

With regards to the regulation of transcription factors, dietary n-3 LC-PUFA significantly decreased the expression of *srebp*-*1* and *lxrα* (*P* < 0.05) (Fig. 5A). The *in vitro* studies clearly showed that both DHA and EPA significantly suppressed the transcription of *srebp*-*1* and *lxrα* (*P* < 0.05) (Fig. 5B and C).

### Transcriptional regulation of the *L*. *crocea* elovl4 and elovl5 by Lxrα and Srebp-1

**Figure 6:**
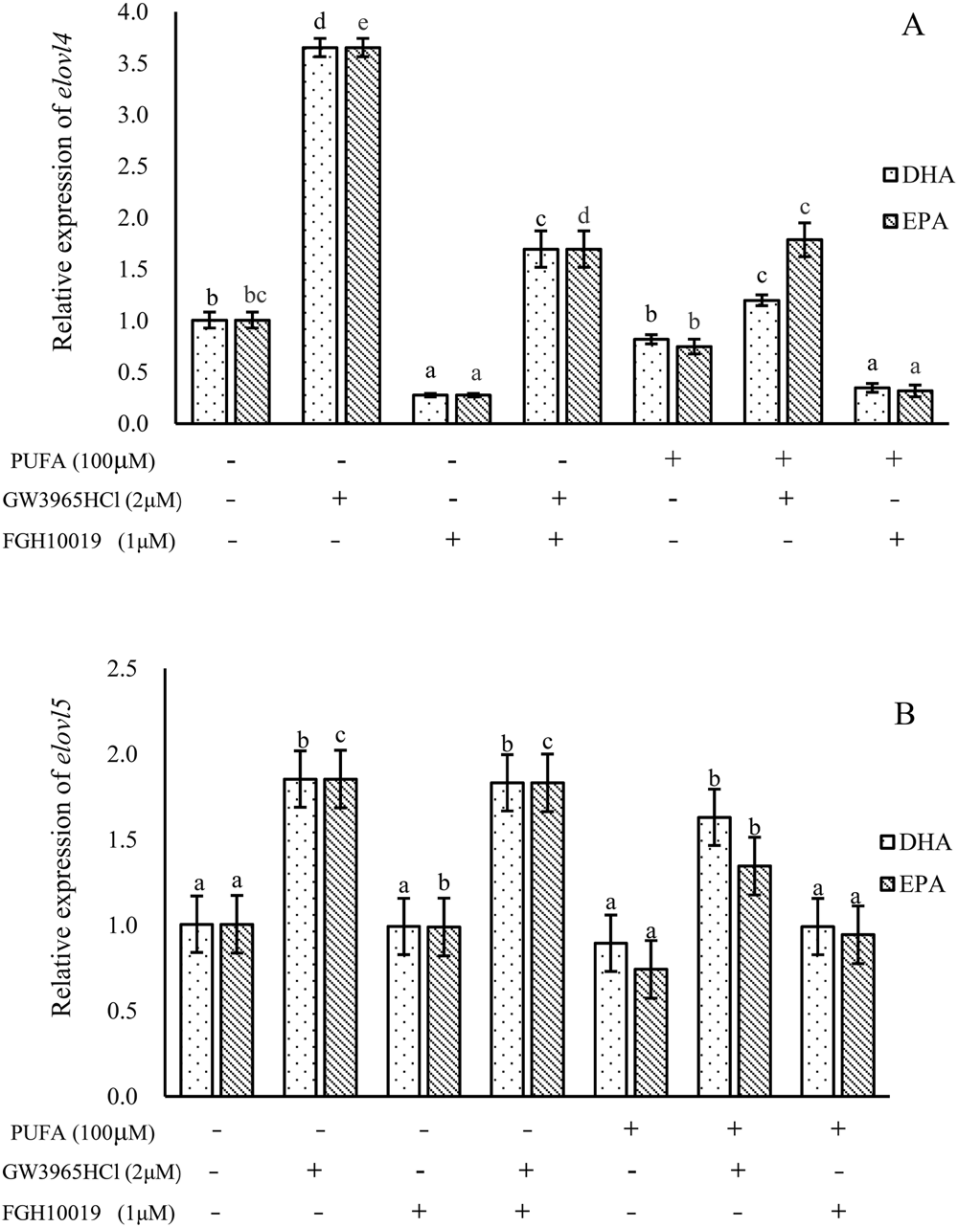
Transcriptional regulation of *L*. *crocea elovl4* (**A**) and *elovl5* (**B**) by Lxrα and Srebp-1. The Lxrα activator, GW3965HCl, and Srebp-1 inhibitor, FGH10019, were used to investigate effects on the expression of *L*. *crocea elovl4* and *elovl5*. “PUFA” indicates that hepatocytes were incubated with either docosahexaenoic acid (DHA) or eicosapentaenoic acid (EPA). Values (means ± standard error of the mean, SEM) in bars that have the same letter are not significantly different (*P* > 0.05; Tukey’s test) among treatments (n = 3).

Over-expression of Lxrα significantly increased the transcription of *elovl4* (*P* < 0.05), but the expression of *elovl4* was significantly suppressed through the inhibition of Srebp-1 (*P* < 0.05) in yellow croaker hepatocytes (Fig. 6A). These results indicated that both *lxrα* and *srebp* up-regulated *elovl4*. The addition of DHA or EPA significantly reduced the abovementioned up-regulation effects of *lxrα* and *srebp* (*P* < 0.05) (Fig. 6A). In addition, the inhibition of Srebp-1 significantly decreased the regulatory role of LXRα on the transcription of *elovl4* (*P* < 0.05) (Fig. 6A).

Regarding the transcription of *elovl5*, over-expression of *lxrα* significantly increased the transcription of *elovl5* (*P* < 0.05), while the inhibition of Srebp-1 had no significant effect on *elovl5* expression (*P* > 0.05) (Fig. 6B). Moreover, the addition of DHA or EPA suppressed the up-regulation of *elovl5* by Lxrα (*P* < 0.05) (Fig. 6B). Nevertheless, inhibition of Srebp-1 did not affect the regulation of *elovl5* by Lxrα (*P* < 0.05) (Fig. 6B).

### Dual-luciferase reporter assays

**Figure 7:**
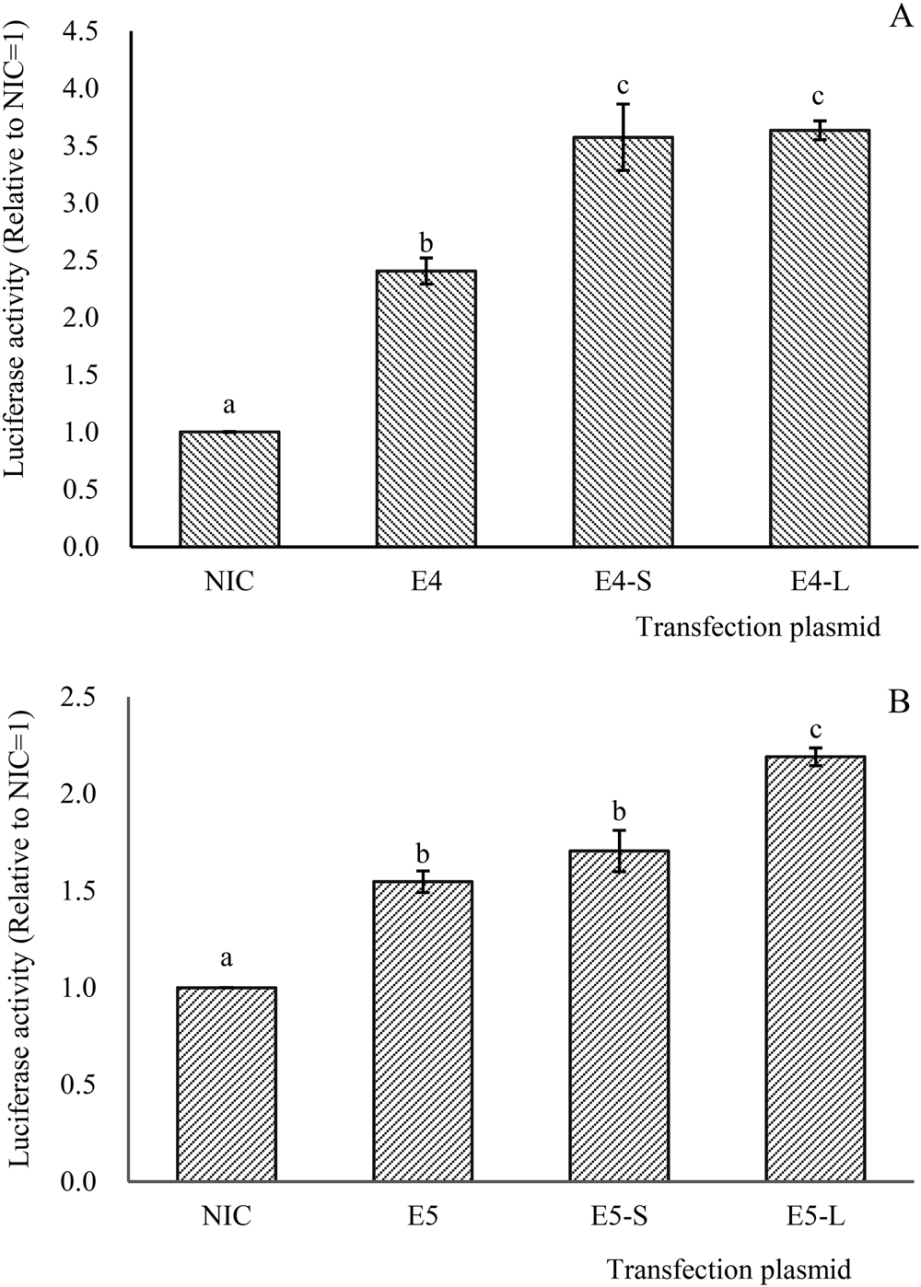
The dual-luciferase reporter assays of *elovl4* (**A**) and *elovl5* (**B**). The type of transfection plasmid applied to each group was as follows: NIC: PGL3 − basic + PCS2 + PRL − CMV; E: PGL-Elo promoter + PCS2 + PRL − CMV; E − S: PGL-Elo promoter + PCS2 − SREBP-1 + PRL − CMV; E − L: PGL-Elo promoter + PCS2 − LXRα + PRL − CMV. Values (means ± standard error of the mean, SEM) in bars that have the same letter are not significantly different (*P* > 0.05; Tukey’s test) among treatments (n = 3).

In order to investigate the molecular mechanisms involved in the regulation of *L*. *crocea elovl4* and *elovl5*, regions of 1921 (GenBank Accession No. KY863452) and 2291 bp (GenBank Accession No. KY863451), respectively, upstream of the initiation codon of the genes were cloned to perform dual-luciferase reporter assays. For *elovl4*, the reporter activity was 2.41-fold than the control (Fig. 7A). The croaker *elovl4* reporter activity was significantly elevated by over-expression of *lxrα* and *srebp*-*1* (Fig. 7A). For *elovl5*, the reporter activity was 1.66-fold than the control (Fig. 7B). Similarly, the *L*. *crocea elovl5* reporter activity was significantly elevated by over-expression of *lxrα* (Fig. 7B). However, the over-expression of *srebp*-*1* had no significant effect on the activity of the *elovl5* promoter (Fig. 7B).

## Discussion

The large yellow croaker is widely cultured in China due to its great commercial value. Recently, a study investigating the effects of FO replacement in diets for *L*. *crocea* showed that low retention of LC-PUFA in muscle occurred, thus reducing the nutritional quality of the product^26^. This was possibly due to the low LC-PUFA biosynthetic ability of large yellow croaker as generally reported for most marine species^27^. Elongases play key roles in the biosynthesis of LC-PUFA in fish^7, 11^ and thus a better understanding of the underlying mechanisms regulating the transcription of elongases would contribute to strategies designed to enhance the endogenous LC-PUFA synthetic ability of the large yellow croaker that may lead to increased nutritional value of farmed products for human consumers.

Seven members of the Elovl protein family have been described in vertebrates^9, 28^. Typically, Elovl2 and Elovl5 play roles in the biosynthesis of LC-PUFA in vertebrates, having somewhat overlapping functions possibly related to their common evolutionary origin^29^. Whereas Elovl5 is present in virtually all teleosts, Elovl2 appears to be lost during evolution and is absent in Acanthopterygii that encompass the majority of farmed marine fish^11^. Fish generally possess two Elovl4-like elongases^11^, but only the so-called “isoform b” has PUFA as preferred substrates^25^. In the present study, we conducted the molecular cloning of an *elovl4* and functionally characterized the functions of the herein reported Elovl4 and the previously cloned *elovl5* ^20^. In addition, we investigated regulatory mechanisms of transcription factors in both elongases and the influence of “dietary” LC-PUFA in both *in vivo* and *in vitro* systems. The results demonstrated that the expression of the two elongases was regulated by n-3 LC-PUFA via specific transcriptional factors that exert different actions on each elongase.

The isolated croaker Elovl4 possessed all the features of the elongase family including transmembrane domains, a histidine box (HXXHH), and an arginine residue and single lysine residue (RXKXX) in the canonical C-terminal, indicating its role in LC-PUFA biosynthesis^30^. Zebrafish possessed two Elovl4 enzymes, Elovl4a and Elovl4b, with different functions^25^. Both the Elovl4 in zebrafish had roles in the biosynthesis of saturated VLC-FA, while Elovl4b was also able to operate on PUFA substrates producing polyenes of up to 36 carbons and thus participated in the biosynthesis of VLC-PUFA^25^. Phylogenetic analysis of the newly cloned *L*. *crocea elovl4* showed that it was an orthologue of zebrafish Elovl4b and not Elovl4a. Consistent with these genes being orthologues of the zebrafish Elovl4b, previously cloned *elovl4*-like elongases isolated from cobia^26^, Atlantic salmon^31^, rabbitfish^32^ and orange-spotted grouper^15^, had similar functions compared to zebrafish Elovl4b and they were all capable of elongating both saturated and polyunsaturated FA. In contrast, the *L*. *crocea* Elovl4-b like elongase did not show any activity towards endogenous saturated FA in yeast and thus does not appear to have a role in the biosynthesis of saturated VLC-FA as its previously characterized counterparts. The reasons why Elovl4 in *L*. *crocea* does not play a role in the biosynthesis of saturated VLC-FA remain uncertain, but a recent study reporting the functional characterization of Elovl4 in nibe croaker *Nibea mitsukurii* did not report any activity towards saturated FA using a similar expression system as that used herein. While it is possible that the activity of the nibe croaker Elovl4 towards endogenous saturated FA in yeast was simply not measured and/or reported, the close phylogenetic relationship between *L*. *crocea* and *N*. *mitsukurii*, both from the Sciaenidae family, possibly indicated that some Elovl4 from certain species or families within teleosts have subfunctionalized during evolution, as has occurred in the fatty acyl desaturase Fads2^33^, a protein family that also plays key roles in LC-PUFA biosynthesis in vertebrates^11, 28^.

In agreement with the functional data obtained with fish Elovl4^15, 26, 31, 32^, the *L*. *crocea* Elovl4 showed activity towards PUFA substrates and thus confirmed its role in the biosynthesis of VLC-PUFA. Thus, the *L*. *crocea* Elovl4 exhibited high elongation efficiency towards a range of PUFA substrates, namely C_20_ (20:5n-3 and 20:4n-6) and C_22_ (22:5n-3 and 22:4n-6) PUFA, with C_18_ substrates (18:4n-3 and 18:3n-3) being elongated to a lesser extent. The functions of VLC-PUFA are not fully understood, but studies in mammals have suggested that these compounds play pivotal roles in vision^34^ and reproduction^35^. Importantly, the large yellow croaker Elovl4 could produce 24:5n-3 from directly supplemented 22:5n-3 or by step-wise elongation of 20:5n-3. Such an ability of Elovl4 has been noted as an important feature by which this enzyme can contribute to DHA biosynthesis through the Sprecher pathway in species that, like most marine farmed species including the large yellow croaker, might have lost Elovl2. Thus, the elongation product of Elovl4, namely 24:5n-3, can be desaturated to 24:6n-3 and subsequently chain shortened to 22:6n-3 by partial β-oxidation^36^ Similar activities were also demonstrated for Elovl4 from zebrafish (isoform b), Atlantic salmon, cobia, rabbitfish, nibe croaker and orange-spotted grouper^15, 25, 31, 32, 37, 38^.

The large yellow croaker Elovl5 could efficiently elongate C_18_ (18:4n-3 and 18:3n-6) and C_20_ (20:5n-3 and 20:4n-6) substrates, although C_22_ PUFA including 22:5n-3 and 22:4n-6 were only elongated to a small degree. Such substrate preference as observed here with the *L*. *crocea* Elovl5 is highly conserved among teleost Elovl5^11^. Furthermore, the *L*. *crocea* Elovl5 was also able to elongate 18:3n-3 and 18:2n-6, FA substrates not only for Δ6 desaturases to initiate LC-PUFA biosynthesis through the classical “Δ6 pathway” but also for elongases like Elovl5 to initiate biosynthesis through the alternative “Δ8 pathway”^7^. The ability of the *L*. *crocea* Elovl5 to actively elongate 18:3n-3 and 18:2n-6 has also been reported in Elovl5 elongases characterized from the southern bluefin tuna (*Thunnus maccoyii*), Japanese eel (*Anguilla japonica*), striped snakehead (*Channa striata*) and orange-spotted grouper (*E*. *coioides*)^14, 16, 39, 40^. While further studies are required to elucidate the desaturase gene and function repertoire, the results from the present study clearly indicated that *L*. *crocea* expressed genes encoding elongases with all the activities required for the biosynthesis of C_20–22_ LC-PUFA from C_18_ precursors supplied in the diet.

Aquafeeds are currently formulated with decreasing levels of the marine ingredients FM and FO due to increased demand and unpredictable availability, leading to high price volatility. Nutritional regulation of genes encoding desaturases and elongases has been proposed as a possible strategy to enhance the endogenous production on n-3 LC-PUFA and optimise their retention in farmed fish^8, 41, 42^. In general, n-3 LC-PUFA can suppress the transcription of the Elovl4 and Elovl5 encoding genes in fish^14–20^. Additionally, previous studies found that LC-PUFA and their metabolites can regulate transcription of lipid metabolism related genes through modulation of transcription factors including, among others, Lxrα and Srebp-1^24^. However, the regulatory mechanisms of these transcription factors on the two herein studied elongases has been barely investigated. It has been demonstrated that n-3 LC-PUFA can suppress the transcription of the *elovl4* and *elovl5* elongases both *in vivo* and *in vitro*, indicating the existence of negative feedback regulation in the LC-PUFA biosynthetic pathway. Moreover, the expression of *srebp*-*1* and *lxrα* were also suppressed by n-3 LC-PUFA both *in vivo* and *in vitro* in the present study, and this may largely account for the feedback suppression of expression of both *elovl4* and *elovl5*.

In mammals, Srebp-1 positively regulates transcription via interacting with sterol response elements (SRE) in the promoter region of target genes^22^. Furthermore, Lxrα can activate transcription directly and/or indirectly via regulating Srebp-1 or other transcription factors^23, 43^. Dual-luciferase reporter assays were conducted in the present study to clarify the regulatory mechanisms by which Lxrα and Srebp-1 modulate the expression of *elovl4* and *elovl5*. The results showed that the large yellow croaker Elovl5 reporter activity was induced by over-expression of *lxrα*, but not by over-expression of *srebp*-*1*. This may indicate that the transcription of *elovl5* was up-regulated by Lxrα directly, but not dependent on Srebp-1. This observation is consistent with the results of studies carried out in Atlantic salmon and orange-spotted grouper^16, 44^. The results of the *in vitro* experiment conducted in the present study to confirm the above findings showed that the expression of *elovl5* in *L*. *crocea* hepatocytes was induced by over-expression of *lxrα*, while inhibition of the expression of *srebp*-*1* had no effect on the expression of *elovl5* and could not repress the activation effect of Lxrα on *elovl5*. This confirmed the direct stimulatory role of Lxrα on *elovl5*, and suggested that such regulatory mechanisms operated differently compared to mammals^24^. Promoter studies showed that the large yellow croaker Elovl4 reporter activity was induced by over-expression of both *lxrα* and *srebp*-*1*. This observation indicated that the expression of the *L*. *crocea elovl4* can be up-regulated through Lxrα and Srebp-1. Additionally, the *in vitro* results showed that the transcription of *elovl4* in hepatocyte was significantly induced by over-expression of *lxrα*, but significantly repressed by inhibiting the expression of *srebp*-*1*. The inhibition of the expression of *srebp*-*1* would significantly repress the activation effect of Lxrα on *elovl4*. These results suggested that Lxrα can promote the expression of croaker *elovl4* via a Srebp-dependent pathway. The above observations indicated differential regulation of Lxrα and Srebp-1 on Elovl4 and Elovl5 in the large yellow croaker. Knowledge of the underlying regulation mechanism of these two elongases can contribute to the development of novel practical approaches to enhance endogenous LC-PUFA production via both nutritional strategies and genetic modification.

In summary, the large yellow croaker Elovl4 exhibited all the structural features of Elovl proteins. Functional studies showed that Elovl4 and Elovl5 of *L*. *crocea* had complementary functions since the latter could effectively elongate both C_*18*_ and C_20_ PUFA substrates, while Elovl4 was more effective in the elongation of C_20_ and C_22_ FA substrates. Moreover, the functional characterization of the *L*. *crocea* Elovl4 demonstrated its role in the biosynthesis of VLC-PUFA. In addition, n-3 LC-PUFA can regulate the transcription of *elovl5* and *elovl4* elongases through Lxrα. Moreover, Lxrα could mediate the transcription of *elovl4* directly or indirectly via regulating the transcription of *srebp*-*1*.

## Materials and Methods

### Cloning and phylogenetic analysis of the *L*. *crocea* full-length *elovl4* cDNA

Total RNA was extracted from isolated croaker liver using Trizol Reagent (Takara, Japan). Thereafter, first strand cDNA was synthesized using Prime Script^TM^ RT reagent Kit (Takara). Two degenerate primers (Elo4-F and Elo4-R) (Supplementary Table) were designed based on highly conserved regions of *elovl4* cDNA sequences of other fish and used for PCR (Eppendorf Mastercycler Gradient, Eppendorf, Hamburg) using PrimeSTAR^®^ HS DNA Polymerase (Takara, Japan). The amplified products were purified (SK8131, Sangon Biotech, Shanghai), ligated into the pEASY-T1 vector (TransGen Biotech, China), transformed into competent Trans1-T1 cells (TransGen Biotech, China) and sequenced (Biosune Biotech, Shanghai, China), followed by blasting on GenBank to confirm its high similarity with other Elovl4 proteins. Gene specific primers (Supplementary Table) were designed to obtain the full-length *elovl4* cDNA sequence by 5′-and 3′-rapid amplification of cDNA ends (RACE) PCR according to the manufacturer’s instructions (SMARTer™ RACE cDNA Amplification Kit, Clontech, CA, USA). RACE PCR products were purified, cloned into pEASY-T1 vector, and sequenced as described above.

The deduced amino acid (aa) sequence of the newly cloned croaker *elovl4* cDNA was aligned with other orthologues including human (*Homo sapiens*, NP_073563), mouse (*Mus musculus*, NP_683743), rat (*Rattus norvegicus*, NP_001178725), zebrafish (*Danio rerio*, NP_957090 and NP_956266), cobia (*Rachycentron canadum*, ADG59898), and Atlantic salmon (*Salmo salar*, NP_001182481). Multiple sequence alignment was performed with Mega 6.0. A phylogenetic tree was constructed on the basis of aa sequences using the neighbor-joining method and including the newly cloned croaker Elovl4 sequence and those from other vertebrate Elovl proteins^45^. Confidence in the resulting phylogenetic tree branch topology was measured by bootstrapping the data through 1000 iterations.

### Functional characterization of the *L*. *crocea* Elovl5 and Elovl4 elongases

PCR fragments corresponding to the open reading frames (ORF) of the Elovl5 and Elovl4 elongases were amplified from croaker liver cDNA, using High Fidelity PrimeScript® RT-PCR Kit with primers containing restriction sites for *Hin*dIII and *Xho*I (Supplementary Table). The DNA fragments containing the croaker *elovl5* or *elovl4* ORF were digested with corresponding restriction endonucleases (Takara, Japan) and then ligated into a similarly restricted pYES2 vector (Invitrogen, UK) to yield the resulting plasmid constructs pYES2-Elo5 and pYES2-Elo4. *Saccharomyces cerevisiae* competent cells were transformed (S.c. EasyComp Transformation Kit, Invitrogen, USA) with the purified recombinant plasmid constructs pYES2-Elo5 or pYES2-Elo4. Transformation and selection of yeast with recombinant plasmids, and yeast culture were conducted as described in detail previously^46, 47^. A single colony of transgenic yeast was grown in *S*. *cerevisiae* minimal medium^−uracil^ supplemented with potential fatty acid (FA) substrates. For Elovl5, each culture was supplemented with one of the following substrates: 18:3n-3, 18:2n-6, 18:4n-3, 18:3n-6, 20:5n-3, 20:4n-6, 22:5n-3 and 22:4n-6. For Elovl4, each culture was supplemented with one of the following substrates: 18:4n-3, 18:3n-6, 20:5n-3, 20:4n-6, 22:5n-3, 22:4n-6 and 22:6n-3. In order to compensate for differences in FA update efficiency by yeast, final concentrations of FA substrates varied according to their fatty acyl chain lengths, 0.5 mM (C_18_), 0.75 mM (C_20_) and 1.0 mM (C_22_)^25^. Elovl4 plays a role in the biosynthesis of very long-chain (>C_24_) saturated FA (VLC-FA)^25^ and thus pYES2-Elo4 transformed yeast were also grown in the absence of exogenously added fatty acids to enable comparison with saturated FA profiles of yeast transformed with the empty pYES2. For both Elovl5 and Elovl4 assays, yeast were grown for 2 days and then harvested, washed twice with 5 mL ice-cold HBSS (Invitrogen, UK) and freeze dried for 24 h for further analyses. Yeast transformed with empty pYES2 were additionally grown in the presence of all PUFA substrates tested in both assays for Elovl5 and Elovl4 as further control treatments.

### Fatty acid analysis

Total lipids were extracted from yeast samples and fatty acids derivatized to methyl esters (FAME) as described in detail previously^48^. FAME were identified and quantified after splitless injection and run with temperature programming in an Agilent 6850 Gas Chromatograph system equipped with a Sapiens-5MS (30 m × 0.25 μm × 0.25 μm) capillary column (Teknokroma, Barcelona, Spain) coupled to a 5975 series MSD (Agilent Technologies, Santa Clara, CA, USA) as described by Monroig *et al*.^27^. The elongation of exogenously added PUFA substrates (18:3n-3, 18:2n-6, 18:4n-3, 18:3n-6, 18:4n-3, 20:5n-3, 20:4n-6, 22:5n-3 and 22:6n-3) was calculated by the step-wise proportion of substrate FA converted to elongated product as [areas of first product and longer chain products/(areas of all products with longer chain than substrate + substrate area)] × 100^15^.

### Animal experiments

The present study was carried out in strict accordance with the recommendations in the Guide for the Use of Experimental Animals of Ocean University of China. The protocols for animal care and handling, methods and experimental procedures used in this study were approved by the Institutional Animal Care and Use Committee of Ocean University of China. A 10-week feeding trial was conducted to investigate the effect of dietary n-3 LC-PUFA on expression of *elovl4* and *elovl5*, and the regulation of certain transcription factors on the two enzymes. DHA-enriched oil (DHA content: 40.6% of total fatty acid in the form of DHA-methyl ester, Jiangsu Tiankai Biotechnology Company Limited, China) and EPA-enriched oil (EPA and DHA at 45.9% and 23.8%, respectively, of total fatty acids both in the form of TAG; Hebei Haiyuan Health Biological Science and Technology Company Limited, China) were utilized in different ratios to form three isoproteic (43% crude protein) and isolipidic (18% crude lipid) diets with low (0.46%), moderate (1.05%) and high (2.44%) n-3 LC-PUFA content (the ratio of DHA to EPA was approximately 2.0). Large yellow croaker were obtained from a local farm located in Xiangshan Bay, Ningbo, China. At the beginning of the experiment, the fish were fasted for 24 h and weighed after being anesthetized with eugenol (1:10,000) (Shanghai Reagent, China). Triplicate groups of croaker (individual weight of 10.0 ± 0.6 g) were distributed into each sea cage (1 m × 1 m × 1.5 m) at a stocking density of 60 individuals, and fed twice daily (05:00 and 17:00 h) to apparent satiation for 70 days. At the end of the experiment, croaker were fasted for 24 h and anesthetized and culled by an overdose of MS222 (ethyl 3-aminobenzoate methanesulfonic acid salt, Aldrich, USA). Livers of three fish per cage were isolated, frozen in liquid nitrogen and stored at −80 °C for subsequent gene expression analysis of elongases (*elovl4* and *elovl5*) and transcription factors (*lxrα* and *srebp*-*1*). In addition, various tissues (eye, brain, testis, heart, liver, kidney, stomach, intestine and muscle) were isolated from nine croaker individuals fed diets with moderate n-3 LC-PUFA levels (1.05%) to investigate the tissue distribution of *elovl4* mRNA.

### Primary hepatocyte isolation and incubation

The above feeding study provided preliminary data on the *in vivo* nutritional regulation of *elovl4* and *elovl5*. To confirm and support these data, an *in vitro* study was conducted to further investigate the role of certain transcription factors on the regulation of *elovl4* and *elovl5* elongases in *L*. *crocea*. Hepatocytes were isolated from five yellow croaker individuals (~50 g) starved for 24 h according to the published protocols^49^ with slight modification^50, 51^. Briefly, croaker were anesthetized with MS 222 and the branchial arch cut followed by immersion in 70% ethanol for 3 min to sterilize the external surface. Liver tissues were excised aseptically and rinsed twice with phosphate buffered saline (PBS, pH 7.4 at 4 °C) supplemented with amphotericin-B (25 μg mL^−1^), streptomycin (100 μg mL^−1^) and penicillin (100 IU mL^−1^). Thereafter, the liver was aseptically minced into 1 mm^3^ pieces, followed by digestion in 0.25% sterile trypsin at room temperature for 15 min. Trypsin was neutralized with Dulbecco’s Modified Eagle Medium (DMEM, Gibco, USA) containing 20% fetal bovine plasma (FBS, Invitrogen, USA). The cell suspension was collected and filtered through sterile 75 μm mesh, followed by centrifugation at low speed (100 g, 5 min) and the supernatant discarded. Isolated cells were washed in red blood cell lysis buffer (Beyotime Institute of Biotechnology, Haimen, China) for 2 min at 4 °C. Cell viability was evaluated using a hemocytometer under an inverted microscope after the cells were stained with 0.4% Trypan Blue. The hepatocytes were re-suspended in DMEM medium containing 1 mM glutamine, 20% FBS, penicillin (100 IU mL^−1^) and streptomycin (100 μg mL^−1^). Cells suspensions with more than 95% cell viability were used for the subsequent experiments.

Croaker primary hepatocyte suspensions were incubated with fatty acid (DHA or EPA) to confirm the influence of n-3 LC-PUFA on the transcription of the elongases (*elovl4* and *elovl5*) and transcription factors (*lxrα* and *srebp*-*1*) *in vivo*. Fatty acid (DHA and EPA, Cayman Chemical Co., USA) was supplemented to cells in the form of BSA/fatty acid complexes that were prepared at 10 mM concentration according to Ou *et al*.^52^ and stored at −20 °C. Additionally, inhibitors/agonists of transcription factors were used to clarify the role of the transcription factors on the regulation of *elovl4* and *elovl5* elongases in *L*. *crocea*. GW3965 HCl (Selleckchem, Shanghai, China) was used as an LXRα agonist whereas FGH10019 (MCE, USA) was used as a SREBP-1 inhibitor, respectively. Cells were seeded in 6-well plates with a density of 2 × 10^6^ viable cells per well in DMEM/F12 (Gibco) containing 20% FBS, 100 U ml^−1^ penicillin and 100 μg ml^−1^ streptomycin, followed by incubation for 24 h. The hepatocytes were then washed and incubated for 1 h in FBS-free DMEM/F12 medium prior to incubation with EPA, DHA and the above inhibitors or agonists in triplicate wells. After incubation, cells were lysed in the wells and harvested for RNA extraction.

### Real-time quantitative PCR (RT-qPCR) analysis

Specific primers for RT-qPCR were designed by Primer Premier 5.0 (Premier Biosoft) based on the cloned nucleotide sequences. The stability of β-actin was verified and confirmed and used as the reference gene^53^. The amplification was performed in a quantitative thermal cycler (Mastercycler EP Realplex, Eppendorf, Germany). The program was 95 °C for 2 min, followed by 40 cycles of 95 °C for 10 s, 57 °C for 10 s, and 72 °C for 20 s. At the end of each reaction, melting curve analysis of amplification products was carried out to confirm that a single PCR product was present in these reactions. The amplification efficiencies of the target and reference genes were determined from the slope of the log-linear portion of the calibration curve and PCR efficiency = 10^(−1/Slope)^ − 1. The expression levels of the target genes were calculated following the 2^−ΔΔCt^ method described by Livak & Schmittgen^54^.

### Cloning of the *elovl4* and *elovl5* promoters

Genomic DNA was extracted from croaker liver using the SQ Tissue DNA Kit (OMEGA, USA) according to the manufacturer’s instructions. Then, the genomic DNA was digested with four restriction enzymes (*Dra*I, *Eco*RV, *Pvu*II and *Stu*I), purified and ligated to Genome Walker Adaptors following the instructions of Universal Genome Walker 2.0 Kit user manual (Clontech, USA). The promoter region of elovl5 and elovl4 was amplified through a nested (two round) PCR combining the kit supplied primers Ap1 and Ap2 with Advantage 2 PCR Kit (Clontech, USA) and the gene specific primers designed on *elovl5* and *elovl4* cDNA (see Supplementary Table for primer details). The conditions for the primary round PCR were: 7 cycles of 25 s at 94 °C, 3 min at 72 °C, followed by 32 cycles of 25 s at 94 °C, 3 min at 65 °C, then with an additional 7 min at 67 °C after the final cycle. The conditions for the secondary round PCR were: 5 cycles of 25 s at 94 °C, 3 min at 72 °C, followed by 20 cycles of 25 s at 94 °C, 3 min at 65 °C, then with an additional 7 min at 67 °C after the final cycle. The PCR products were purified, cloned into pEASY-T1 and sequenced as described above.

### Expression and reporter plasmids constructs

For expression plasmids, PCR fragments corresponding to the ORF of the croaker *srebp*-*1* (GenBank accession number: KP342262) were amplified with primers containing restriction sites for *Eco*RI and *Xho*I, respectively, and the ORF of croaker *lxrα* (GenBank accession number: XM_019273432) was amplified with primers containing restriction sites for *Cla*I and EcoRI (Supplementary Table). The DNA fragments were digested with corresponding restriction endonucleases (Takara) and ligated into a similarly restricted pCS2^+^ vector (Invitrogen, USA) to yield the expression plasmid constructs pCS2-srebp1and pCS-lxrα. The reporter plasmid Elovl5-Luc and Elovl4-Luc were engineered to contain a fragment of the large yellow croaker Elovl5 and Elovl4 promoters cloned into the *Kpn*I/*Xho*I sites of the pGL3 basic vector (Promega, USA) containing the coding sequences of firefly luciferase cDNA. The renilla luciferase plasmid pRLTK (Promega) was used as an internal control. Plasmids for transfection were prepared using TransGen Plasmid Mini Kit (Beijing, China) according to the manufacturer’s instructions.

### Cell culturing, transfection and luciferase assay

HEK 293 T cells were cultured in DMEM (Gibco) supplemented with 10% fetal bovine plasma (Invitrogen) at 37 °C in a humidified incubator under 5% CO_2_. For DNA transfection, cells were seeded in 24-well plates until they were 90–100% confluent at the time of transfection. Then plasmids were transfected by using the Lipofectamine^TM^ 2000 Reagent (Invitrogen) according to the manufacturer’s instruction. Briefly, 0.6 μg expression plasmid, 0.2 μg reporter gene plasmid, 0.02 μg pRLTK renilla luciferase plasmid, and 2.0 μL Lipofectamine^TM^ 2000 were co-transfected into the cells in each well in the 24-well plate. All assays were performed with three independent transfections. Firefly and renilla luciferase activities were measured using Dual Luciferase Reporter Assay System (Promega, USA) according to the manufacturer’s instructions. Briefly, after 48 h transfection, HEK293T cells in the 24-well plates were washed twice with 100 μL PBS, then lysed with 100 μL 1x passive lysis buffer (PLB) at room temperature for 10 min. Cell lysate (20 μL) was transferred to a clean plate and 50 μL of luciferase assay reagent II and 1x Stop & Glo substrate were added in sequence, then firefly and renilla luciferase activities were measured using a InfiniTE200 plate reader (Tecan, Switzerland).

### Statistical analysis

The results were presented as means ± standard error of the mean (SEM). Data from each treatment were subjected to one-way analysis of variance (ANOVA) and correlation analysis where appropriate using SPSS 19.0 for Windows. Tukey’s multiple range test was chosen as a multiple comparison test with a significance level of 0.05. For the Elovl4 functional characterization, the saturated VLC-FA profiles from yeast expressing the *elovl4* were compared to the control by a Student’s *t*-test (*P* < 0.05).

## Electronic supplementary material

supplementary table
