# Supplementary material for: Functional characterization and differential nutritional regulation of putative Elovl5 and Elovl4 elongases in large yellow croaker (Larimichthys crocea)
Source: Sci Rep. 2017 May 23;7:2303. doi: 10.1038/s41598-017-02646-8 (PMC5442133; doi:10.1038/s41598-017-02646-8)
Supplement: Supplementary file 1 — supplementary table [file 41598_2017_2646_MOESM1_ESM.doc]

**Functional characterization and differential nutritional regulation of putative Elovl5 and Elovl4 elongases in large yellow croaker (*****Larimichthys crocea*)**

**Songlin Li^a^, Óscar Monroig^b^, Tianjiao Wang^a^, Yuhui Yuan^a^,** **Juan Carlos** **Navarro^c^, Francisco Hontoria^c^, Kai Liao^a^, Douglas R. Tocher^b^, Kangsen Mai^a^, Wei Xu^a^, Qinghui Ai^a^***

*^a^Key Laboratory of Aquaculture Nutrition and Feed, Ministry of Agriculture, Ocean University of China, Qingdao 266003, People’s Republic of China.*

*Key Laboratory of Mariculture, Ministry Education of China, Ocean University of China, Qingdao 266003, People’s Republic of China.*

*^b^**Institute of Aquaculture, School of Natural Sciences, University of Stirling, Stirling FK9 4LA, Scotland, UK*

*^c^**Instituto de Acuicultura Torre de la Sal (IATS-CSIC), Ribera de Cabanes 12595, Castellón, Spain*

*Corresponding author

Tel./Fax: +86 532 82031943

E-mail address: qhai@ouc.edu.cn (Q. Ai).

**Supplementary Table**. Sequences of the PCR primers used in this study.

| Primer | Sequence (5’-3’) | Purpose |
| --- | --- | --- |
| Elo4-F | AGACAAGAGGGTGGAGAAMT | RT primer |
| Elo4-R | AGGACAATGAAGGTGACRGC | RT primer |
| Elovl4-F1 | GCACCAAAAAATGATTGTCCACCG | 5’ RACE primer(inner) |
| Elovl4-F2 | GTGACAGCATAGCCAATAAGAACCCA | 5’ RACE primer(outer) |
| Elovl4-R1 | TTCCTGGACACAGTCTTTTTCATCA | 3’ RACE primer (inner) |
| Elovl4-R2 | TGGGTTCTTATTGGCTATGCTGTC | 3’ RACE primer (outer) |
| E4-hindIII-F | CCCAAGCTTATGGAGGCTGTAACACAT | Functional characterization |
| E4-XhoI-R | CCGCTCGAGTTACTCCCTTTTCGCT | Functional characterization |
| E5-hindIII-F | CCCAAGCTTATGGAGACCTTCAATCAT | Functional characterization |
| E5-XhoI-R | CCGCTCGAGTCAATCCACCCTCAGTTTC | Functional characterization |
| E4-sp1 | TCCTGAGCGTAAAGGGCTGGCGGT | Cloning of promoter |
| E4-sp2 | CCAGAGTGGGGAGGGGAGATGACA | Cloning of promoter |
| E5-sp1 | GCCCCATCCACACGATCACAAGGTAC | Cloning of promoter |
| E5-sp2 | GAGCAGTAGCCATCCCCGCACACGC | Cloning of promoter |
| E4p-KpnI-F | CGGGGTACCAGTCATAGATTTTCACTTGC | Construction of reporter plasmid |
| E4p-XhoI-R | CCGCTCGAGGGCTCTGCCTTTGGTCT | Construction of reporter plasmid |
| E5p-KpnI-F | CGGGGTACCGTGTGTCCCCTTACTTCTAT | Construction of reporter plasmid |
| E5p-XhoI-R | CCGCTCGAGTTGTCACCTAGAAAGAGAAC | Construction of reporter plasmid |
| SRE-EcorI-F | CCGGAATTCATGAACAGCCTGTC | Construction of expression plasmid |
| SRE-XhoI-R | CCGCTCGAGCTAGCTGTTGGTGAC | Construction of expression plasmid |
| LXR-CIaI-F | CCATCGATATGTCCACGCTGTCTGT | Construction of expression plasmid |
| LXR-EcoRI-R | CGGAATTCTCACTCGTTGACATCCCAG | Construction of expression plasmid |
| SREBP-F | TCTCCTTGCAGTCTGAGCCAAC | RT-qPCR |
| SREBP-R | TCAGCCCTTGGATATGAGCCT | RT-qPCR |
| LXR-F | TCTCAAAAGGAATGAACGAC | RT-qPCR |
| LXR-R | TAAGGTTGCTGTAGCCTCTCA | RT-qPCR |
| Elovl4-F | GGGCTCTTATTGGCTATGCT | RT-qPCR |
| Elovl4-R | TGCTTCTTCCCGTTATCCTC | RT-qPCR |
| Elovl5-F | ATCACCTTCCTTCACATCTATCACC | RT-qPCR |
| Elovl5-R | GAGGCACCGAAGTACGAATGG | RT-qPCR |
| βactin-F | ctacgagggttatgccctgcc | RT-qPCR |
| βactin-R | tgaaggagtaaccgcgctctgt | RT-qPCR |
